# Item distribution and inter-rater reliability of the German version of the quality of life in Alzheimer’s disease scale (QoL-AD) proxy for people with dementia living in nursing homes

**DOI:** 10.1186/s12877-018-0834-z

**Published:** 2018-06-19

**Authors:** Martin Nikolaus Dichter, Eva-Maria Wolschon, Christian G. G. Schwab, Gabriele Meyer, Sascha Köpke

**Affiliations:** 1 German Center for Neurodegenerative Diseases (DZNE), Stockumer Straße 12, 58453 Witten, Germany; 2 School of Nursing Science, Witten/Herdecke University, Stockumer Straße 12, 58453 Witten, Germany; 3 Institute of Social Medicine and Epidemiology, University of Lübeck, Ratzeburger Allee 160, 23562 Lübeck, Germany; 4 Institute for Health and Nursing Science, Medical Faculty, Martin Luther University, Halle-, Wittenberg, Germany

**Keywords:** Quality of life, Nursing homes, Dementia, Psychometric properties, Reliability

## Abstract

**Background:**

The Quality of Life in Alzheimer’s disease scale (QoL-AD) is a widely used Health Related Quality of Life (HRQoL) instrument. However, studies investigating the instrument’s inter-rater reliability (IRR) are missing. This study aimed to determine the item distribution and IRR of the German proxy version of the QoL-AD (13 Items) and a nursing home-specific instrument version (QoL-AD NH, 15 Items).

**Methods:**

The instruments were applied to 73 people with dementia living in eight nursing homes in Germany. Individuals with dementia were assessed two times by blinded proxy raters. The IRR analyses were based on methodological criteria of the quality appraisal tool for studies of diagnostic reliability (QAREL), the COSMIN group and the single-measure Intra-Class Correlation Coefficient (ICC) for absolute agreement ≥0.70.

**Results:**

All items for both instrument versions demonstrated acceptable item difficulty, with the exception of one item (QoL-AD proxy). The IRR was moderate for the QoL-AD (ICC: 0.65) and insufficient for the QoL-AD NH (ICC: 0.18). The additional computation of the average measure ICC for two proxy-raters demonstrated a strong IRR (ICC: 0.79) for the QoL-AD and a weak IRR for the QoL-AD NH (ICC: 0.31). The detailed analysis of the IRR for each item underpinned the need for the further development of both instruments.

**Conclusions:**

The unsatisfactory IRRs for both instruments highlight the need for the development of a user guide including general instructions for instrument application as well as definitions and examples reflecting item meaning. Priority should be given to the development of reliable proxy-person versions of both instruments.

**Trial registration:**

ClinicalTrials.gov: NCT02295462, Date of registration: 11–20-2014.

## Background

Health Related Quality of Life (HRQoL) has become an important outcome in dementia research [1, 2]. HRQoL is defined as a “multidimensional concept that reflects the individual’s subjective perception of the impact of a health condition on everyday living” [3]. The Quality of life in Alzheimer’s Disease (QoL-AD) scale has been widely used to measure HRQoL in people with dementia [4–7]. The QoL-AD has been developed in the US [4] and is based on the conceptual work by Lawton [8] who defines the QoL concept as multidimensional, including subjective (e.g., perceived QoL and psychological well-being) and objective components (e.g., behavioral competence and environment). The instrument had originally been developed as self-rating version for community-dwelling people with dementia but has also been used frequently as proxy-instrument in the nursing home setting [9–11].

In addition to the self- and proxy version of the QoL-AD, Edelman et al. [5] have developed the QoL-AD NH, an adapted version of the original instrument particularly for people living in nursing homes. Whereas self-rating means that the QoL of a person with dementia is rated by herself/himself, a proxy-rating is defined as the QoL rating of a particular person with dementia by a proxy e.g. relative or caregiver of the person with dementia. According to Pickard & Knight [12] the proxy-rating perspectives “proxy-proxy” and “proxy-person” have been distinguished. While in the former perspective the proxy rates the HRQoL of a person with dementia from his/her proxy perspective, in the latter perspective a proxy assesses the QoL of a person with dementia as he/she thinks the person with dementia would rate him or herself [12]. Both proxy perspectives are appropriate for the assessment of HRQoL. Unfortunately, the applied proxy perspective mostly remains unclear in the literature [13]. One recent study investigated both perspectives (proxy-proxy and proxy-person), comparing them to self-reports of people with and without dementia in nursing homes. The three perspectives were assessed with different versions of the EuroQol-5D [13]. The results show that both proxy-perspectives overestimate low self-reports and underestimate high self-reports. The tendency to attenuate self-ratings existed for both proxy-perspectives with a smaller perspective gap between self-ratings and the proxy-person perspective [13]. These results highlight the need for a clear definition and description and the psychometric investigation of both proxy perspectives. In general, there is little information on the psychometric properties of the proxy versions of the QoL-AD and QoL-AD NH [1, 2]. In particular, there is a lack of information about the inter-rater reliability (IRR) of the QoL-AD proxy and QoL-AD NH proxy scales [2]. This lack of information is often neglected in the literature [1, 14–18] and its impact on the validity of the QoL-AD proxy and QoL-AD NH proxy scales is unclear [19].

A detailed user guide with instructions for the instrument application is available for the self-rating versions, but not for proxy-rating versions. It is particularly unclear whether the items of both proxy versions have to be rated from a proxy-proxy or a proxy-person perspective.

QoL-AD proxy and QoL-AD NH proxy are frequently applied which is most likely due to the instruments’ anticipated feasibility. Thus, the low number of items (QoL-AD proxy = 13 Items, QoL-AD NH proxy = 15 Items) and the fact that no comprehensive training is necessary (instructions for the proxy versions are not available) allows a resource-saving data collection in contrast to other QoL measures for people with dementia (e.g. QUALIDEM, Dementia Care Mapping instrument). The number of missing values has been described as low [9, 11, 20].

The discrepancy between this lack of knowledge and the uncontrolled usage of the QoL-AD proxy and QoL-AD NH proxy emphasizes the relevance of a comprehensive evaluation of the IRR of both measures.

While the German version of the QoL-AD proxy has been available for some years, we have only recently conducted a cross-cultural adaption of the QoL-AD NH proxy to the German context [21].

Based on the international lack of knowledge concerning the IRR of the QoL-AD proxy and QoL-AD NH proxy as well as different perspective gaps between self-ratings and proxy perspectives the objective of the present study was to evaluate item distribution and IRR of both instruments, based on a proxy-proxy perspective for the QoL-AD proxy and a proxy-person perspective for the QoL-AD NH proxy.

## Methods

### Study design

This study was conducted between June 2015 and March 2016 as a nested cohort study within the randomized controlled trial EPCentCare [22], which aimed to reduce antipsychotic medication in nursing home residents. EPCentCare was carried out in three German regions, whereas the present evaluation took place in Northern Germany only. The study sample consisted of people with dementia from eight nursing homes located in Schleswig-Holstein and Hamburg. The investigation of the IRR of the QoL-AD proxy and QoL-AD NH proxy was based on the criteria of the quality appraisal tool for studies of diagnostic reliability (QAREL) [23] and the COSMIN group [24] (e.g. sample size calculation, description of blinding of raters, description of raters).

### Sample size calculation

The sample size calculation was based on an estimated intra-class correlation coefficient (ICC) of 0.75, ratings of two independent raters (registered nurses and nursing assistants) and a width of 0.20 for the 95% Confidence Interval (CI). This resulted in a calculated sample of 75 residents with dementia [25].

### Procedures

According to the inclusion criteria of the EPCentCare trial, nursing homes with at least 50 residents were eligible for participation in the study. For this IRR evaluation the predefined inclusion criteria for residents with dementia was a Dementia Screening Scale score ≥ 3 [26]. Exclusion criteria were a temporary stay in respite care or a primary diagnosis of schizophrenia or bipolar disorders.

Inclusion criteria for caregivers were at least half-time work and at least one year nursing training (“nursing assistant” qualification). Qualification levels of proxy-raters depended on organizational conditions and staffing levels at the time of data collection in the participating nursing homes. Additionally, caregivers had to have been at work on at least seven days within the last two weeks prior to data collection and had to have a close relationship with the assessed resident. Based on these criteria, caregivers were identified and assigned to the assessed residents by the management staff (head nurse) of each participating nursing home. The close relationship with the assessed resident enabled the caregivers to rate the HRQoL based on a proxy-person and proxy-proxy perspective.

The IRR evaluation was based on independent proxy-ratings from registered nurses or nursing assistants referring to the preceding two weeks. Both independent ratings for one measure and one resident took place in the same shift and under the same circumstances. Each caregiver was blinded to the ratings of the other rater. To ensure blinding of raters and standardized data collection, application of QoL-AD proxy and QoL-AD NH proxy was supervised by researchers of the EPcentCare trial, who had been trained in applying QoL instruments, prior to the data collection. The supervision included a short instruction about the assessed construct (e.g. HRQoL, agitated behavior), the underlying time frame (preceding two weeks) and the perspective (e.g. proxy-person). Based on different organizational conditions and staffing levels at the time of data collection, independent ratings by different qualified caregivers (registered nurses and nursing assistants) were possible. During the data collection occasion one caregiver assessed one QoL-AD proxy version at the beginning and one at the end of the data collection. The order of the QoL-AD proxy application was randomly applied.

### Instruments

The QoL-AD proxy consists of 13 items which can be answered by self-rating (person with dementia) or proxy-rating (e.g. caregiver). Response options for all items are “poor”, “fair”, “good” and “excellent” resulting in item scores between 1 to 4 and total scores between 13 and 52, with higher scores indicate higher HRQoL. In 2005 the QoL-AD was translated into German language [27]. For this investigation the German version provided by Mapi Research Trust, Lyon was used [28]. Although information related to the linguistic validation of the German version is not available, in two recent studies, the German QoL-AD proxy demonstrated sufficient internal consistency and structural validity [6, 29].

For the nursing home version (QoL-AD NH proxy), two items of the original version concerning financial and marital status (*Money, Marriage*) were removed and four items added (*People who work here, Ability to take care of oneself, Ability to live with others, and Ability to make choices in one’s life*) [5]. The response options for the QoL-AD NH correspond to the original version, resulting in total scores from 15 to 60. In 2016 the nursing home version was translated into German and linguistically validated [21]. While no information on psychometric properties are available for the German version, the original US proxy-proxy version showed sufficient internal consistency in three studies [5, 30, 31] and a nearly perfect IRR [31].

Cognitive impairment of participating residents was assessed by nursing staff using the Dementia Screening Scale (DSS) [26], a seven item measure with a three point response scale (0, 1, 2) resulting in scores between 0 and 14, with higher scores indicating more cognitive impairment.

Agitated behavior was assessed by nursing staff using a adapted German version [32] of the Cohen-Mansfield Agitation Inventory (CMAI) [33, 34], a proxy-measurement consisting of 25 items rated on a seven-point scale of frequency of occurrence resulting in scores between 25 and 175 [35], with higher scores indicating higher frequencies of agitation. Age, gender, length of stay in nursing home in months, and care dependency level (0, I, II, or III) as defined by the German long-term care insurance, were taken from the residents’ case files. In addition, proxy-rater characteristics were assessed with single items.

### Data analysis

Sample characteristics and item distribution were computed using descriptive statistics. Based on the item distribution the item difficulty was proven. An item mean in the first 20% of the scale was defined as floor effect and in the last 20% as ceiling effect. To gain comprehensive information on the degree of IRR for the QoL-AD proxy and QoL-AD NH proxy, a reliability coefficient was calculated for each measurement item and all measurement items in total. This procedure was based on earlier IRR studies and allowed a detailed interpretation and comparison of the IRR of each item [36, 37]. The IRR for each item was based on a calculation of the overall proportion of agreement (p_o_). Moreover, we computed the linear weighted κ statistics for ordinal data (κ_w_) [38, 39], because p_o_ ignores the possibility that agreement could occur only by chance and instead considers only crude agreement. The two paradoxical properties of κ statistics were considered during the interpretation of the results [40]. The interpretation of κ_w_ values was based on the following ranges: 0.00–0.20, slight; 0.21–0.40, fair; 0.41–0.60, moderate; 0.61–0.80, substantial; and 0.81–1.00, nearly perfect [41]. The IRR of the QoL-AD proxy and QoL-AD NH proxy in total were evaluated using Intraclass Correlation Coefficients (ICC) based on a two-way random-effects model for absolute agreement. Additionally, the average-measure ICC for two raters was calculated. This coefficient estimates the IRR of a collaborative QoL-AD proxy respectively QoL-AD NH proxy rating by two raters. Based on the recommendation by Terwee et al. [24], we targeted κ_w_ and ICC values ≥0.7. To analyze the level of uncertainty, 95% CIs for ICCs and κ_w_ values were examined. The 95% CI for κ_w_ values was based on 10,000 bootstrapped samples and determined using the percentile method [42], which included using the 0.025 and 0.975 percentile levels of the estimated Kappa distributions as interval limits. Statistical analysis was performed using R Version 3.2.4 [43] and the software packages “irr” Version 0.84 [44] and “boot” Version 1.3–18 [45, 46].

## Results

### Characteristics of the sample

The sample consisted of 73 residents with dementia and 21 caregivers from eight nursing homes (Table 1).

**Table 1:** Characteristics of the sample

Characteristics		
People with dementia	n	= 73
Age in years^a^	87.38	(±7.71)
Female^a^	38	(71.7%)
Length of stay in nursing home in months^a^	34.57	(±31.14)
Care dependency levels^a, b^		
• None	0	(0.0%)
• considerable (= I)	9	(17.0%)
• severe (= II)	25	(47.2%)
• most severe (≥ III)	19	(35.8%)
Dementia Screening Scale (DSS)	9.01	(±3.14)
• mild or moderate dementia (3–7)	28	(38.4%)
• severe dementia (8–14)	45	(61.6%)
Challenging behaviour (CMAI)^a^	34.87	(±10.48)
Caregivers (proxy-raters)	n	= 8 ^c^
Age in years	34.75	(±13.76)
Female	8	(100.0%)
Registered nurses	5	(62.5%)
Working experience in years	9.38	(±5.63)
Working hours per week	38.75	(±3.54)
Experience in proxy-ratings		
• no	6	(75.0%)
• yes, at least once	1	(12.5%)
• yes, regularly (≥ twice a month)	1	(12.5%)

Data are reported as means (±SD) or absolute number (%)

^a^These results were based on *n* = 53 people with dementia, because these characteristics were missing for *n* = 20 people of the total sample

^b^As determined by expert raters of the medical service of the statutory long-term care insurance system

^c^The analyzed data includes answers of 21 raters. For 13 proxy-raters there are no data available

### Item distribution

The descriptive investigation of the QoL-AD proxy items (proxy-proxy perspective) demonstrated a balanced distribution (Table 2). The response option “good” was used most frequently whereas the response category “excellent” was used least often. Distributions of the other response options were balanced. Only one item showed a floor effect (item 10, ability to do chores around the house).

**Table 2:** Item distribution based on two observations for each case – German version of the QoL-AD proxy and QoL-AD NH proxy

QoL-AD proxy (proxy-proxy perspective), *n* = 73									QoL-AD NH proxy (proxy-person perspective), n = 73								
No.	Item	response options				descriptive			No.	Item	response options				descriptive		
How do you judge …		poor	fair	good	excellent	missing	mean (±SD)		How do you think the resident would judge his/her …		poor	fair	good	excellent	missing	mean (±SD)	
		1	2	3	4						1	2	3	4			
1.	… the physical health of the resident?	27	41	67	11	0	2.42	(±0.88)	1.	… physical health?	39	49	47	11	0	2.21	(±0.92)
2.	… the energy of the resident?	37	39	63	7	0	2.27	(±0.90)	2.	… energy?	29	54	59	4	0	2.26	(±0.81)
3.	… the mood of the resident?	25	62	55	4	0	2.26	(±0.77)	3.	… mood?	27	55	53	10	1	2.32	(±0.86)
4.	… the living situation of the resident?	4	23	103	16	0	2.90	(±0.61)	4.	… living situation?	23	44	70	9	0	2.45	(±0.83)
5.	… the memory of the resident?	72	55	17	2	0	1.65	(±0.74)	5.	… memory?	27	38	67	14	0	2.47	(±0.90)
6.	… the family of the resident?	36	28	45	37	0	2.57	(±1.12)	6.	… family?	23	25	59	37	2	2.76	(±1.01)
7.	… the marriage of the resident?	55	14	39	37	1	2.40	(±1.23)									
8.	… the friends of the resident?	61	37	35	8	5	1.93	(±0.95)	7.	… friends?	34	27	51	25	9	2.49	(±1.06)
9.	… his−/herself as a whole?	4	63	72	7	0	2.56	(±0.63)	8.	… -self overall?	10	44	72	20	0	2.70	(±0.79)
10.	… the ability of the resident to do chores around the house?	93	40	13	0	0	1.45 ^a^	(±0.65)	9.	… ability to keep busy?	21	46	69	9	1	2.46	(±0.82)
11.	… the ability of the resident to do things for fun?	78	36	29	3	0	1.71	(±0.86)	10.	… ability to do things for fun?	21	64	55	6	0	2.32	(±0.77)
12.	… the money of the resident?	13	38	35	18	42	2.56	(±0.92)									
									11.	^b^… people who work here?	12	25	92	16	1	2.77	(±0.75)
									12.	… ability to take care of him−/herself?	43	38	54	11	0	2.23	(±0.96)
									13.	… ability to live with others?	19	58	62	7	0	2.39	(±0.77)
									14.	… ability to make choices in his/her life?	26	41	62	17	0	2.48	(±0.92)
13.	… life as a whole?	8	69	66	1	2	2.42	(±0.61)	15.	… life as a whole?	13	60	67	6	0	2.45	(±0.72)
Frequencies of the response options and missing’s in total	n	513	545	639	151	50			Frequencies of the response options and missing’s in total	n	367	668	939	202	14		
	%	27.76	29.49	34.58	8.17	2.63				%	16.87	30.70	43.15	9.28	0.64		
Qol-AD proxy score	1 Rating					25	30.13	(±5.72) ^c^	Qol-AD NH proxy score	1 Rating					7	38.76	(±7.84) ^c^
	2 Rating					24	30.08	(±5.76) ^c^		2 Rating					4	35.27	(±7.11) ^c^

^a^Item with floor effect, i.e. mean in the first 20% of the scale (< 1.6)

^b^The question begins with: ‘How satisfied do you think the residents feels about the …’

^c^Only observations without any missing value were included (Qol-AD proxy: *n* = 39; Qol-AD NH proxy: n = 62)

Analyses for the QoL-AD NH proxy (proxy-person perspective) yielded similar results. However, no item showed floor or ceiling effects.

Missing value analyses demonstrated low percentages of missing values in general. Only item 12 (Money) of the QoL-AD proxy showed a high percentage of missing values (29%). The main reason for this was nurses’ refusal to rate or a lack of knowledge to assess residents’ financial situation. A descriptive comparison of the elven comparable items shows no clear pattern that one perspective or QoL scale lead to higher QoL ratings.

### Inter-rater reliability

The results of the IRR evaluation for the QoL-AD proxy and the QoL-AD NH proxy are displayed in Table 3. Based on the high percentage of missing values for item 12 of the QoL-AD proxy two ICC values based on different sample were computed. Both resulting ICC values (ICC: 0.65 = 13 items, ICC: 0.63 = 12 items) demonstrated a moderate IRR for the QoL-AD proxy. The average-measure ICC for two proxy-raters demonstrated a strong IRR of 0.79 (13 items) or 0.77 (12 items) for the QoL-AD proxy. In contrast, the IRR results for the Qol-AD NH proxy were weak: (0.18 or 0.31 for the average-measure ICC).

**Table 3:** Inter-rater reliability results for the German version of the QoL-AD proxy (proxy-proxy perspective) and QoL-AD NH proxy (proxy-person perspective)

QoL-AD poxy	(*n* = 39) ^f^	ICC (95% CI):	0.65 [0.79	(0.42–0.80) ^a^ (0.60–0.89)] ^b^	QoL-AD NH proxy	(*n* = 62) ^f^	ICC (95% CI):	0.18 [0.31	(− 0.05–0.40) ^a^ (− 0.10–0.57)] ^b^
	(without item 12, *n* = 65)	ICC (95% CI):	0.63 [0.77	(0.45–0.76) ^a^ (0.62–0.86)] ^b^					
No.	How do you judge …	p_o_^c^	κ_w_	(95% CI) ^d^	No.	How do you think the resident would judge his/her …	p_o_^c^	κ_w_	(95% CI) ^d^
1.	… the physical health of the resident?	0.49	0.36	(0.19–0.51)	1.	… physical health?	0.44	0.30	(0.12–0.46)
2.	… the energy of the resident?	0.45	0.32	(0.16–0.47)	2.	… energy?	0.37	0.08	(−0.09–0.25)
3.	… the mood of the resident?	0.51	0.33	(0.18–0.48)	3.	… mood?	0.35	0.11	(−0.07–0.28)
4.	… the living situation of the resident?	0.59	0.19	(0.05–0.34)	4.	… living situation?	0.29	−0.03	(−0.16–0.11)
5.	… the memory of the resident?	0.49	0.21	(0.03–0.39)	5.	… memory?	0.44	0.16	(−0.01–0.33)
6.	… the family of the resident?	0.52	0.55	(0.41–0.67)	6.	… family?	0.39	0.30	(0.13–0.46)
7.	… the marriage of the resident?	0.47	0.37	(0.20–0.53)					
8.	… the friends of the resident?	0.40	0.19	(0.01–0.36)	7.	… friends?	0.30	0.11	(−0.07–0.28)
9.	… his−/herself as a whole?	0.49	0.15	(−0.03–0.32)	8.	… -self overall?	0.44	0.05	(−0.09–0.2)
10.	… the ability of the resident to do chores around the house?	0.56	0.23	(0.08–0.38)	9.	… ability to keep busy?	0.38	0.07	(−0.10–0.24)
11.	… the ability of the resident to do things for fun?	0.42	0.18	(0.02–0.33)	10.	… ability to do things for fun?	0.36	−0.01	(−0.19–0.18)
12.	… the money of the resident?	0.33	0.07	(−0.11–0.25)					
					11.	^f^… people who work here?	0.43	0.11	(−0.06–0.27)
					12.	… ability to take care of him−/herself?	0.33	0.12	(−0.04–0.27)
					13.	… ability to live with others?	0.42	0.16	(0.00–0.31)
					14.	… ability to make choices in his/her life?	0.36	0.11	(−0.04–0.27)
13.	… life as a whole?	0.63	0.36	(0.19–0.53)	15.	… life as a whole?	0.55	0.38	(0.19–0.53)

^a^Intra-class correlation coefficient and corresponding 95% CI

^b^Average-measure ICC for two raters in parentheses and corresponding 95% CI

^c^Overall proportion of agreement (the ratio of exact agreement between raters)

^d^Linear weighted Kappa values. CIs were based on 10,000 bootstrapped samples

^e^Item with floor effect, the mean is in the first 20% of the scale (< 1.6), see Table 2

^f^Complete case analysis

For the QoL-AD proxy, κ_w_ values ranged from 0.07 (item 12: money) to 0.55 (item 6: family). The p_o_ ranged between 0.33 (item 12: money) and 0.63 (item 13: life as a whole). IRR results for the QoL-AD NH proxy items ranged between − 0.03 (item 4: living situation) to 0.38 (item 15: live overall) for the κ_w_ and between 0.29 (item 4: living situation) to 0.55 (item 15: live overall) for the p_o._

## Discussion

### Item distribution

This study describes a comprehensive evaluation of the item distribution and inter-rater reliability of the German QoL-AD proxy and QoL-AD NH proxy.

The investigation of the item distribution of the QoL-AD proxy demonstrated a balanced distribution of the four response options. Twelve out of 13 items showed an acceptable item difficulty. Only one item (item 10: ability to do chores around the house) showed a floor effect and item 12 (Money) showed a high percentage of missing values (29%). One reason for these results for item 10 and 12 might be a missing cross-cultural adaption of the QoL-AD proxy for the German context and in particular for German nursing homes.

The analysis for the QoL-AD NH proxy yielded similar results with an acceptable item difficulty for all 15 items. With the exception of the identified floor effect for item 10 of the QoL-AD proxy, these descriptive results are in the line with previous results [4, 5, 21, 47]. Given the relatively small sample size, the identified floor effect for the item 10 of the QoL-AD proxy must be interpreted with caution.

A descriptive comparison of the eleven comparable items shows no clear pattern that one perspective leads to higher QoL ratings. Thus, compared to the comparable items rated in both perspectives, we identified higher mean values for the items 1, 2 and 4 of the QoL-AD (proxy-proxy) and for the items 3, 5, 6, 7, 8, 9, 10 and 15 of the QoL-AD NH (proxy-person). We assumed systematically higher QoL ratings based on a proxy-person perspective compared to a proxy-proxy perspective. This assumption was based on previous studies, which demonstrated systematically lower proxy-based QoL ratings compared to self-ratings [48, 49], and on one recent study, which showed a smaller perspective gap between self-ratings and the proxy-person perspective [13]. The major reason for the missing difference between both perspectives might be the only moderate to weak IRR of the applied scales and perspectives.

### Inter-rater reliability

The IRR results demonstrate a moderate IRR for the QoL-AD proxy (ICC: 0.65, 13 items, ICC: 0.63, 12 items) and an insufficient IRR for the QOL-AD NH (ICC: 0.18). The additional computation of the average measure ICC for two proxy-raters demonstrated a strong IRR of 0.79 (13 items) or 0.77 (12 items) for the Qol-AD proxy and a weak ICC for the QoL-AD NH proxy (0.31).

The detailed analysis of the IRR of each item yielded heterogeneous results. Based on κ_w_ and p_o_, only item 6 (family) of the QoL-AD proxy demonstrated a moderate IRR. All other QoL-AD proxy items showed fair (items 1: physical health, 2: energy, 3: mood, 5: memory, 7: marriage, 10: ability to do chores around the house, 13: life as a whole) or slight IRR (items: 4: living situation, 8: friends, 9: his−/herself as a whole, 11: ability to do things for fun, 12: money). Based on the high number of missing values and the slight IRR of item 12 (money of the resident) we recommend the exclusion of this item for the QoL-AD proxy application in nursing homes.

For the QoL-AD NH proxy only items 1 (physical health) and 15 (life overall) yielded a fair IRR. All other items demonstrated a slight IRR.

The in-depth analysis of the IRR indicates the need for improvement of both instruments for their application in research and practice. An improvement of the IRR might be reached through a structured instrument user guide including clear definitions and examples related to the meaning of each QoL-AD proxy and QoL-AD NH proxy item. The positive effect of such a user guide has been demonstrated in a recent IRR study on the dementia-specific QoL instrument QUALIDEM [37].

This study is the first IRR study dealing with the proxy version of the QoL-AD. Therefore, study results can only be compared to the IRR results of other dementia-specific QoL and HRQoL instruments [2]. The IRR of the Alzheimer Disease-Related Quality of Life (ADRQL) instrument was evaluated in two studies showing different results. While the US version of the ADRQL demonstrated excellent IRR with ICC values between 0.90 and 1.0 [31], a Swiss study identified slight to moderate ICC values depending on ADRQL subscales (Social interaction: ICC = 0.36, Awareness of self, ICC: 0.61, Feelings and mood, ICC: 0.13. Enjoyment of activities, ICC: 0.02, Response to surrounding,: ICC: − 0.17) [50]. Both study results have to be interpreted with caution because of several methodological limitations [2]. The Quality of Life in Late stage Dementia scale (QUALID) demonstrated good IRR with an ICC value of 0.83 [51]. For the instrument QUALIDEM a recent study yielded a good IRR for all subscales for people with mild to very severe dementia (ICC = 0.79–0.96) with the exception of the subscale negative affect (ICC = 0.64) [37]. These three instruments reflect overall QoL. One frequently used instrument for the assessment of dementia-specific HRQoL is the DEMQOL [17]. Unfortunately, no information were available on IRR of the DEMQOL proxy-version which would have allowed a comparison to our study results [2]. Due to the heterogeneous range of QoL domains assessed with different QoL and HRQoL instruments, a comparison of IRR results between different instruments is limited. However, IRR results of instruments like QUALIDEM and QUALID demonstrate that good IRR values are achievable for proxy-rated dementia-specific QoL.

Our IRR results for the QoL-AD NH proxy can be compared to one previous US study which demonstrated a high IRR with a ICC value of 0.99 [31]. In contrast, our IRR results seem low. However, the study by Sloane et al. [31] had several methodological limitations and a proxy-proxy perspective. In contrast, our results are based on a proxy-person perspective. The different perspectives also explain the differences between the Qol-AD proxy and the QoL-AD NH proxy in our study. A comparison of equal items (item 1–6 and 7–10) from the QoL-AD proxy and QoL-AD NH proxy show lower p_o_. and κ_w_ for the items assessed with the proxy-person perspective (QoL-AD-NH proxy).

The QoL-AD proxy data were based on a proxy-proxy perspective which means that proxies rate the HRQoL of care recipients from their proxy perspective. This might be an easier perspective compared to the so-called proxy-person perspective as there is no need for a perspective shift by the proxy rater. Nevertheless, proxy-proxy ratings can also be influenced by attitudes [52], life satisfaction, the assessment circumstances and challenging behaviors of people with dementia living in nursing homes [53]. Moreover, HRQoL ratings based on proxy-proxy ratings might not be particularly valid due to the partial loss of subjectivity of the HRQoL assessment [54].

In contrast, the proxy-person perspective requires a change of perspectives. Here, a proxy assesses the HRQoL of a person with dementia as he/she thinks the person with dementia would rate him or herself. It can be assumed that this perspective is more difficult for proxies, thus the required level of individuality for each HRQoL rating is high.

The results of a recent study [13], which showed a smaller perspective gap between self-ratings and the proxy-person perspective, underpin the need for the further development of the proxy-versions of the QoL-AD and QoL-AD NH to enable the assessment of an IRR proxy-person perspective for both instruments.

### Limitations

This study is the first investigation of IRR of the QoL-AD based on a proxy-proxy perspective and the QoL-AD NH based on a proxy-person perspective. The strengths of this IRR study are the in-depth analysis of the IRR of each instrument item, the inclusion of people at all stages of dementia and the methodological rigor based on the criteria of the quality appraisal tool for studies of diagnostic reliability (QAREL) as well as the COSMIN group [24]. The following limitations should be considered when interpreting the results.

First, although the preplanned sample sizes were almost achieved, the identified width of CIs of the computed ICC values range between 0.29 and 0.67. Especially when interpreting the IRR of the QoL-AD NH this statistical uncertainty has to be taken into account. Our results provide a good basis for sample size calculations of further IRR studies.

Second, the applied data collection procedure led to a QoL-AD proxy and a QoL-AD NH proxy assessment for a resident with dementia by one caregiver on one occasion. Despite the random order of the ratings a possible influence of the rating of one measure on the rating of the second measure cannot be excluded.

Third, proxy-raters’ characteristics have to be interpreted with caution due to the high rate of missing values.

Fourth, a close relationship between the proxy and the assessed person with dementia will help proxy raters to reach this required level of individuality. Usually primary models of nursing care are used in German nursing homes. The head nurse was instructed to assign a primary nurse to the assessed residents. Unfortunately, the shortage of nurses in German nursing homes may have influenced the assignment of the head nurses. A varying understanding of this criterion by the assigning head nurse may be jointly responsible for the IRR results.

## Conclusions

This IRR study demonstrated a moderate IRR for the QoL-AD based on a proxy-proxy perspective and an insufficient IRR for the QoL-AD NH (proxy-person perspective). According to established cut off points for the interpretation of IRR values there is a need for the improvement of both instruments. We recommend the development of a user guide including general instructions for the application of both instruments as well as definitions and examples reflecting the meaning of each item. Priority should be given to the development of reliable proxy-person versions of both instruments. Until a user guide is available, the QoL-AD proxy might be conducted as a collaborative rating by at least two proxy-raters. However, this approach is limited to a proxy-proxy perspective which means partial loss of the subjectivity of the HRQoL assessment and as a result, a reduction in validity. Additionally, we recommend the exclusion of item 12 (money of the resident) for the QoL-AD proxy application in nursing homes.

The QoL-AD NH based on a proxy-person perspective should not be used until a user guide is available and further IRR studies have demonstrated an improved IRR.

## Abbreviations

95% CI: 95% Confidence Interval
ADRQL: Alzheimer Disease-Related Quality of Life
CMAI: Cohen-Mansfield Agitation Inventory
COSMIN: COnsensus-based Standards for the selection of health Measurement Instruments
DSS: Dementia Screening Scale
EPCentCare: Effect of person-centred care on antipsychotic drug use in nursing homes
HRQoL: Health Related Quality of Life
ICC: Intra-class correlation coefficient
IRR: Inter-rater reliability
p_o_: proportion of agreement
QAREL: Quality Appraisal tool for studies of diagnostic RELiability
QoL-AD NH: Quality of life in Alzheimer’s Disease Nursing Home; QoL: Quality of Life
QoL-AD: Quality of life in Alzheimer’s Disease
QUALID: Quality of Life in Late stage Dementia scale
κ_w_: weighted kappa statistics
